# A randomized comparison of two prophylaxis regimens and a paired comparison of on-demand and prophylaxis treatments in hemophilia A management

**DOI:** 10.1111/j.1538-7836.2011.04611.x

**Published:** 2012-03

**Authors:** L A VALENTINO, V MAMONOV, A HELLMANN, D V QUON, A CHYBICKA, P SCHROTH, L PATRONE, W-Y WONG, THE PROPHYLAXIS STUDY GROUP

**Affiliations:** * Hemophilia and Thrombophilia Center, Rush University Medical CenterChicago, IL, USA; † Department of Reconstructive Orthopedic Surgery for Hemophilia Patients, Hematology Research Center under the Russian Academy of Medical Sciences (RAMS)Moscow, Russia; ‡ Department of Hematology and Transplantology, Medical University of GdańskPoland; § Hemophilia Treatment Center, Orthopaedic HospitalLos Angeles, CA, USA; ¶ Departments of Pediatric Bone Marrow Transplantation, Oncology, and Hematology, Wroclaw Medical UniversityWroclaw, Poland; ** Baxter Healthcare CorporationWestlake Village, CA, USA

**Keywords:** factor VIII, hemophilia A, previously treated patients, prophylaxis

## Abstract

*Background:* Prophylaxis with factor (F)VIII is considered the optimal treatment for managing hemophilia A patients without inhibitors. *Objectives:* To compare the efficacy of two prophylaxis regimens (primary outcome) and of on-demand and prophylaxis treatments (secondary outcome), and to continue the evaluation of immunogenicity and overall safety of the ADVATE Antihemophilic Factor (Recombinant), Plasma/Albumin Free Method (rAHF-PFM). *Patients/Methods:* Previously on-demand-treated patients aged 7–59 years (*n* = 66) with FVIII levels ≤ 2% received 6 months of on-demand treatment and then were randomized to 12 months of either standard (20–40 IU kg^−1^ every other day) or pharmacokinetic (PK)-tailored (20–80 IU kg^−1^ every third day) prophylaxis, both regimens intended to maintain FVIII trough levels at or above 1%. Efficacy was evaluated in terms of annualized bleeding rates (ABRs). As subjects were first treated on-demand and then on prophylaxis, statistical comparisons between these treatments were paired. *Results:* Twenty-two (33.3%) subjects on prophylaxis experienced no bleeding episodes, whereas none treated on-demand were free from an episode of bleeding. ABRs for the two prophylaxis regimens were comparable, whereas differences between on-demand and either prophylaxis were statistically significant (*P* < 0.0001): median (interquartile range [IQR]) ABRs were 43.9 (21.9), 1.0 (3.5), 2.0 (6.9) and 1.1 (4.9) during on-demand treatment, standard, PK-tailored and any prophylaxis, respectively. There were no differences in FVIII consumption or adverse event rates between prophylaxis regimens. No subject developed FVIII inhibitors. *Conclusions:* The present study demonstrates comparable safety and effectiveness for two prophylaxis regimens and that prophylaxis significantly reduces bleeding compared with on-demand treatment. PK-tailored prophylaxis offers an alternative to standard prophylaxis for the prevention of bleeding.

## Introduction

Hemophilia A is an X-chromosome-linked recessive genetic disorder caused by defective or deficient plasma factor (F)VIII and consequently insufficient coagulant activity, resulting in hemorrhages of variable degrees. Patients with severe disease (FVIII levels < 1% of normal) are at risk of spontaneous bleeding into joints, muscles and internal organs, as well as trauma-induced bleeding after injury and surgery. Repeated bleeding into joints, which may occur as frequently as 20–30 times per year, is a major cause of morbidity and leads to clinically significant hemophilic arthropathy [1].

Prophylactic therapy with FVIII is considered to be the optimal treatment for patients without inhibitors and is aimed at reducing the number of hemorrhages [2–4]. While much of the evidence supporting prophylaxis is observational, previous studies have confirmed the efficacy of prophylaxis in children in preventing or delaying arthropathy by focusing on joint outcomes and spontaneous hemarthroses [3,5–8]. In addition, the early initiation of prophylaxis may have a protective effect against inhibitor development, the most serious complication associated with FVIII treatment [9,10]. In adults who have already developed hemophilic arthropathy, prophylaxis is aimed at slowing progression of joint deterioration and improving mobility, and thus, quality of life [11,12].

As part of a comprehensive clinical program for ADVATE Antihemophilic Factor (Recombinant), Plasma/Albumin Free Method (rAHF-PFM), this clinical study compares the effectiveness of two prophylactic treatment regimens, as well as between on-demand and prophylaxis treatments, in preventing bleeding in hemophilia A patients. As earlier observations indicated that patients with FVIII levels between 1% and 5% have less frequent bleeding than those with levels < 1% [13], both prophylaxis regimens were intended to maintain FVIII levels at or above 1%. One regimen (standard prophylaxis) was based on common practice with every other day dosing [4,14] and the other (PK-tailored prophylaxis) was customized for each individual based on pharmacokinetics (PK) with every third day dosing. As bleeding prevention has been well described in young children [15], the present study focused on older patients with existing joint disease in whom prophylaxis was not recently practiced. This is the first study designed to generate prospective data for stringent comparisons of bleeding rates.

## Clinical study methods

### Patients

The present study was conducted in compliance with international Good Clinical Practice, and national and local regulatory requirements, and is registered at ClinicalTrials.gov (NCT00243386). The protocol was approved by the ethics committees of participating institutions, and written consent was obtained from patients before enrollment.

The main inclusion criteria were an age > 7 and under 65 years, a clinical diagnosis of moderately severe to severe hemophilia A (baseline FVIII level ≤ 2% of normal), on-demand treatment for at least 12 months with at least 150 exposure days to FVIII concentrates, at least eight joint hemorrhages before enrollment and a negative HIV status, or if positive, with a stable CD4 count ≥ 400 cells mm^−3^. The main exclusion criteria were a history of FVIII inhibitor (titer ≥ 0.6 BU [Bethesda unit]), detectable FVIII inhibitors at screening (titer ≥ 0.4 BU), chronic liver disease, immunodeficiency, another hemostatic defect and the need for major surgery.

### Study design

This was an open-label, multicenter study with two comparisons. The first was a randomized, two-arm, parallel comparison of two prophylaxis regimens, and the second was a longitudinal, non-randomized, cross-over comparison of on-demand and prophylaxis treatments. The primary endpoint was differences in annualized bleeding rates (ABRs) between the two prophylaxis regimens. Secondary endpoints included differences in ABRs between subjects first treated on-demand and then on prophylaxis, differences in weight-adjusted rAHF-PFM annual consumption, efficacy of rAHF-PFM for the control of bleeding, differences in health-related quality of life (HRQoL), and the continued assessment of rAHF-PFM immunogenicity, safety and toxicity.

**Fig 1:**
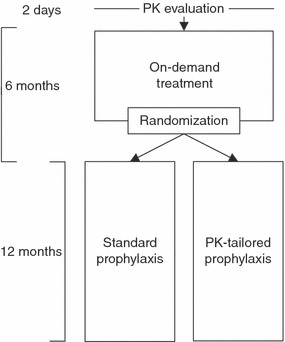
Study design.

The study design is displayed in Fig. 1. Upon enrollment and after at least a 72-h washout period, subjects received 50 ±5 IU kg^−1^ of rAHF-PFM by intravenous (i.v.) bolus at a maximum infusion rate of 10 mL min^−1^ for a PK evaluation (Recommendations of the ISTH FVIII/IX Scientific Standardization Committee for Evaluation of New or Investigational Clotting Factor Concentrates and CPMP guidelines.). Next, subjects received 6 months of on-demand treatment with dosing dependent on the severity and type of bleeding episode (as described in the Supporting Information). After completing the on-demand treatment period, subjects were randomized to receive 12 months of either standard or PK-tailored prophylaxis treatment. The randomization sequence was created using sas version 8.2 (Cary, NC, USA), stratified by 0, 1–2 or ≥ 3 target joints (defined as a joint in which ≥ 4 hemorrhages occurred within a period of 6 months, or > 20 lifetime hemarthroses [16]) with a 1:1 allocation to treatment regimens using a random block size of 2, and provided to the investigator via an automated assignment system as the subject neared completion of on-demand treatment.

### Treatments

Standard prophylaxis dosing was between 20–40 IU kg^−1^ every 48 ±6 h by i.v. bolus with the particular dosage determined by the investigator. PK-tailored prophylaxis dosing was 20–80 IU kg^−1^ every 72 ±6 h by i.v. bolus with the dosage determined at the PK evaluation for each subject based on the following formula: *D = (*2^72/*t*^*)*/*r*; where *D* was dose (IU kg^−1^), 72 was the infusion interval (h), *t* was the estimated terminal half-life and *r* was the incremental recovery. Dose adjustments were permitted for standard prophylaxis within the allowable range according to clinical circumstances, and for PK-tailored prophylaxis if a subject experienced ≥ 2 bleeding episodes during their last 3-month study period, exhibited FVIII trough levels < 1% at the 3-month visit and was FVIII-inhibitor free.

Throughout the study, bleeding was treated according to routine clinical practice. For bleeding episodes occurring during the prophylaxis period, subjects resumed their regimen on the next scheduled day after the last infusion for treatment.

### Pharmacokinetic, clinical and quality-of-life assessments

The PK evaluation included 10 sampling time points up to 48 h postinfusion. FVIII activity had to have decreased monotonically from 1 h postinfusion until pre-infusion values were approached. Terminal half-life, incremental recovery (using the maximal concentration) and clearance were determined as described previously [17,18]. Once the prophylaxis period began, FVIII trough levels were assessed every 3 months.

Descriptions of bleeding episodes (including etiology, severity and anatomical site[s]) were recorded in subject diaries and verified by the investigator. Each bleeding episode may have included more than one anatomical site and the episode was categorized as a joint type if any bleeding site(s) occurred in a joint; otherwise (if no bleeding sites were in joints), the event was categorized as a non-joint type. Hemostatic efficacy was assessed by the number of infusions used to treat each episode and the subject’s rating based on a four-point ordinal scale (excellent, good, fair or none; full descriptions are provided in the Supporting Information) [5].

FVIII inhibitor assessments were performed every 3 months after a minimum 48-h washout period, using the Nijmegen modification of the Bethesda assay [19]. Adverse events (AEs) were recorded in subject diaries and verified by the investigator. Complete blood count and clinical chemistry tests were performed every 3 months, and clinically significant events were reported as AEs.

Subjects ≥ 14 years of age completed a HRQoL questionnaire (SF-36v1 [20]) at screening and after each treatment period.

### Statistical analyses

The sample size assumed an ABR variance of at least that observed for compliant subjects in a previous study [21], and thus, 30 subjects per prophylaxis regimen (60 in total) would detect a difference of 2.5 bleeding episodes per year between the two prophylaxis regimens. To account for approximately 10% attrition, at least 66 subjects were planned for enrollment.

Efficacy analyzes were performed with two analysis sets: (i) intention-to-treat (ITT) which included subjects who completed at least one study visit and (ii) per-protocol (PP) which included subjects who had > 90% of the predicted number of infusions and no major protocol deviations. For the two prophylaxis regimens, a square root transformation of the ABRs (*X*′ = √[*X* + 0.5]) allowed a comparison using a parameteric, paired *t*-test (primary endpoint). Median differences of ABRs and percentage reductions of ABRs between treatment regimens were evaluated using the non-parametric Wilcoxon’s signed-rank test with each test performed at a 5% alpha level and adjusted for multiple testing (0.05 ÷ number of tests), with no *P*-value > 0.01 considered statistically significant. The comparisons between on-demand and prophylaxis were paired as each subject was first treated on-demand and then on prophylaxis. To further assess differences in ABRs between on-demand and prophylaxis treatments, a negative binomial mixed model was used to evaluate the number of bleeding episodes as the dependent variable with the logarithm of time as the offset variable and regimen as the independent variable. A repeated effect over time was included because each subject contributed multiple observations during each treatment period.

HRQoL health domain scores, physical component scores (PCS) and mental component scores (MCS) were compared between the two prophylaxis regimens and between on-demand and prophylaxis treatments using 10 two-sided, paired Wilcoxon’s signed-rank tests with all performed at an alpha level of 5% and adjusted for multiple comparisons. The minimal important difference (MID) for the SF-36 instrument has been estimated at three points. Therefore, differences of three points or more were used to interpret whether statistically significant differences measured using SF-36 were clinically meaningful.

Annualized FVIII consumption and rates of treatment-related AEs for each prophylaxis period were compared using a Mann–Whitney *U*-test.

## Results

### Subjects

**Fig 2:**
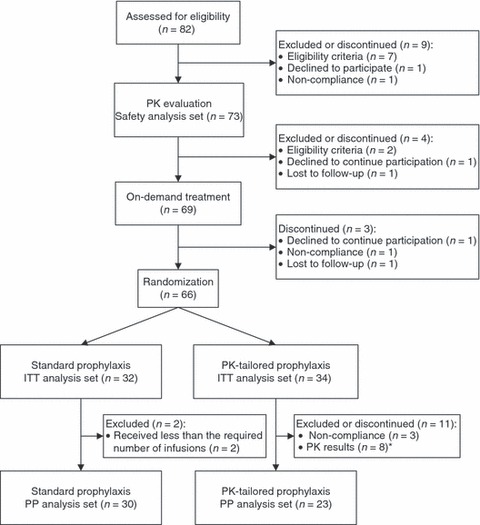
Subject disposition flow diagram.

Of 82 enrolled subjects at nine US and 21 European sites between January 2006 and June 2010, 73 received at least one dose of rAHF-PFM. Sixty-six subjects completed the 6-month on-demand period and were randomized to a 12-month prophylaxis period (32 on standard and 34 on PK-tailored prophylaxis). These 66 subjects comprised the ITT analysis set, and of these, 53 (30 on standard and 23 on PK-tailored prophylaxis) comprised the PP analysis set. For details on subject disposition refer to Fig. 2.

All treated subjects were male and their median age was 26 years, ranging from 7 to 59 years. Overall subject baseline characteristics are presented in Table 1; the characteristics were similar between prophylaxis treatment arms and the differences in median age and hemophilia severity were not statistically significant. PK parameters (half-life, incremental recovery and clearance) are provided in Table 2.

**Table 1:** Subjects’ characteristics

	No. (%) of subjects treated:			
	
	All (73 subjects)	On-demand* (66 subjects)	Standard prophylaxis* (32 subjects)	PK-tailored prophylaxis* (34 subjects)
Age				
Median; range; IQR† in years	26; 7–59	27.5; 7–59; 20	31.5^‡^; 10–55; 22	24.5^‡^; 7–59; 13
≥ 7 to < 16 years (%)	9 (12.3)	9 (13.6)	4 (12.5)	5 (14.7)
≥ 16 years (%)	64 (87.7)	57 (86.4)	28 (87.5)	29 (85.3)
Race				
White (%)	64 (87.7)	58 (87.9)	30 (93.8)	28 (82.4)
Hispanic (%)	4 (5.5)	3 (4.5)	0	3 (8.8)
Black or African American (%)	3 (4.1)	3 (4.5)	2 (6.3)	1 (2.9)
Asian (%)	1 (1.4)	1 (1.5)	0	1 (2.9)
Other (%)	1 (1.4)	1 (1.5)	0	1 (2.9)
Severity of hemophilia				
Severe (%)	63 (86.3)	58 (87.9)	30 (93.8)‡	28 (82.4)‡
Moderately severe (%)	10 (13.7)	8 (12.1)	2 (6.3)‡	6 (17.6)‡
Number of target joints				
None (%)	3 (4.1)	3 (4.5)	1 (3.1)	2 (5.9)
1–2 (%)	26 (35.6)	24 (36.4)	12 (37.5)	12 (35.3)
≥ 3 (%)	44 (60.3)	39 (59.1)	19 (59.4)	20 (58.8)

* Intention-to-treat analysis set.

† IQR, interquartile range calculated for treatment periods only.

‡ Differences between prophylaxis regimens were not statistically significant. PK, pharmacokinetic.

**2:** Mean (± SD) pharmacokinetic parameters

	≥ 14 years		< 14 years
		
PK parameter	ITT (65 subjects)	PP (57 subjects)	ITT and PP (six subjects)
Terminal half-life (h)	13.91 (5.07)	13.95 (5.30)	14.66 (5.21)
Incremental recovery (IU dL^−1^ × IU kg^−1^)	1.81* (0.41)	1.85* (0.40)	1.49 (0.27)
Clearance (mL [kg × h]^−1^)	3.89 (1.21)	3.91 (1.18)	5.17 (1.94)

ITT, intention-to-treat analysis set; PP, per-protocol analysis set.

* Geometric mean.

The mean (range) length of treatment periods were 185 (137–245) days for on-demand, 362 (283-307) days for standard prophylaxis and 344 (97–394) days for PK-tailored prophylaxis; total subject study days were 12 241, 11 571 and 11 711, respectively (ITT analysis set). Over the course of these treatment periods, median (range) doses per infusions were 30.7 (5.1–119.4), 31.4 (11.8–80.9) and 43.0 (13.0–107.1) IU kg^−1^ for on-demand, standard and PK-tailored prophylaxis treatments, respectively. For subjects on PK-tailored prophylaxis, the actual dose reflected the pre-determined dose, as the ratio of the pre-determined to actual dose ranged from 0.9 to 1.2. Throughout the prophylaxis period, target FVIII levels were maintained: median (range) trough levels were 3.0 (0.5–45.0) IU dL^−1^ during standard prophylaxis (56 observations in 24 subjects) and 1.0 (0.5–10.0) during PK-tailored prophylaxis (52 observations in 23 subjects).

### Prevention of bleeding

A total of 1640 bleeding episodes occurred in 66 of 66 subjects during the on-demand period, 104 episodes occurred in 19 out of 32 subjects during standard prophylaxis and 141 episodes in 25 out of 34 subjects during the PK-tailored prophylaxis. Thus, none of the subjects treated on-demand were bleeding episode-free during the 6-month period; whereas, when these same subjects were switched to prophylaxis, 13 out of 32 and 9 out of 34 treated on standard and PK-tailored prophylaxis, respectively, (33.3% overall) experienced no bleeding episodes during the 12-month prophylaxis treatment period. There were 25 bleeding episodes involving a gastrointestinal site, one of which included an intracranial site; all were deemed mild or moderate by the investigator and none were major or life-threatening.

### Primary endpoint

Efficacy in terms of ABRs was compared between the two prophylaxis regimens. There was no difference in mean (± standard deviation [SD]) transformed ABRs: 1.6 ± 1.2 for the 32 subjects who were treated on standard prophylaxis and 1.9 ± 1.1 for the 34 subjects on PK-tailored prophylaxis (*P* = 0.2588; ITT analysis set). Similarly, there was no difference in median (interquartile range [IQR]) ABRs: 1.0 [3.5] and 2.0 [6.9] for standard and PK-tailored prophylaxis, respectively (*P* = 0.1467; ITT analysis set).

### Secondary endpoints

**Fig 3:**
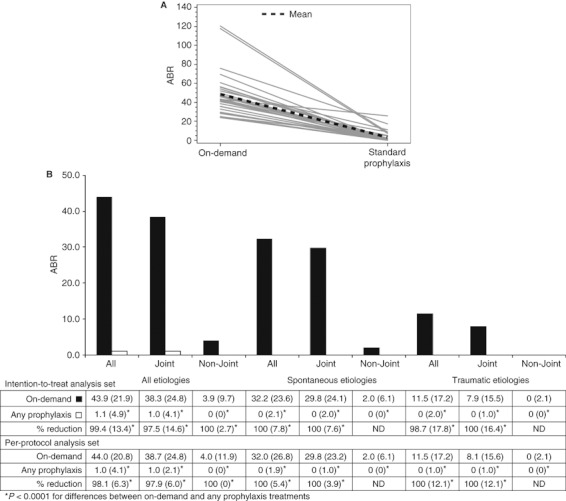
Comparison of annualized bleeding rates (ABRs). (A) Mean ABRs for each subject during treatment regimens (intention-to-treat [ITT] analysis set). (B) Median (interquartile range [IQR]) ABRs and percentage reductions during on-demand and any prophylaxis treatments. ND, not determined.

Comparisons between on-demand and any prophylaxis treatment are shown in Fig. 3. Median (IQR) ABRs were 43.9 (21.9) for the 66 subjects treated on-demand, compared with 1.0 (3.5) when 32 subjects were switch to standard prophylaxis and 2.0 (6.9) when 34 subjects were switched to PK-driven prophylaxis, or 1.1 (4.9) when any of 66 subjects were switched from on demand to any prophylaxis (ITT analysis set). The relative reduction in median ABR was 99.4% for subjects treated with any prophylaxis compared with during on-demand treatment. As expected, these data demonstrate a highly statistically significant (*P* < 0.0001) reduction in ABR while on any prophylaxis compared with during on-demand treatment. ABRs for all types of bleeding (joint and non-joint) and etiologies (spontaneous and traumatic) were also significantly lower (*P* < 0.0001) on any prophylaxis compared with during on-demand treatment. These reductions in ABRs were similar for subjects on standard or PK-tailored prophylaxis regimens compared with on-demand treatment (see Supporting Information).

To further confirm these results, a negative binomial mixed effects model was used to evaluate the influence of treatment regimen on the number of bleeding episodes over time. As expected, a decreased incidence of bleeding was associated with prophylaxis treatment (coefficient −2.865, *P* < 0.0001).

Although the present study was not designed to assess the impact of adherence with the prophylaxis regimen on clinical outcome, we performed a *post hoc* analysis defining an ‘adherent’ subject as one who received ≥ 90% of the prescribed number of infusions. Sixty-one (of 66) subjects were considered ‘adherent’ (30 on standard and 31 on PK-tailored prophylaxis), whereas two on standard prophylaxis and three on PK-tailored prophylaxis were ‘non-adherent.’ Median (IQR) ABRs for ‘non-adherent’ subjects were 14.0 (23.8) for standard and 6.9 (10.4) for PK-tailored prophylaxis compared to ABRs for ‘adherent’ subjects of 1.0 (2.1) and 2.0 (4.1), respectively (ITT analysis set; by definition, none of the subjects in the PP analysis set were ‘non-adherent’).

### Treatment of bleeding

The proportions of bleeding episodes treated with 1, 2, 3 or ≥ 4 infusions were similar across all treatments, as were the proportion of bleeding treatments rated excellent, good, fair and none. The majority of bleeding episodes were treated with one infusion and the majority of treatments were rated by the subject as having an excellent or good hemostatic efficacy response (Table 3).

**Table 3:** Hemostatic efficacy of the treatment of bleeding

	On-demand		Standard prophylaxis		PK-tailored prophylaxis		Any prophylaxis	
				
	ITT	PP	ITT	PP	ITT	PP	ITT	PP
Percentage of hemorrhages by number of infusions used for treatment								
1	72.0	71.1	73.1	74.2	64.7	63.5	68.1	68.6
2	17.1	18.2	12.9	7.6	26.6	29.7	21.1	19.3
3	7.9	7.9	4.3	6.1	3.6	2.7	3.9	4.3
≥ 4	3.1	2.8	9.7	12.1	5.0	4.1	6.9	7.9
Percentage of hemorrhages by rating of response to treatment^*^								
Excellent	32.7		41.9		23.7		31.0	
Good	56.4		40.9		54.0		48.7	
Fair	10.0		17.2		7.9		11.6	
None	0.2		0		14.4		8.6	
Unknown	0.8		0		0		0	

ITT, intention-to-treat analysis set; PP, per-protocol analysis sets. ^*^Described in the Supporting Information.

### rFVIII usage

There was no difference in median (IQR) annualized rAHF-PFM consumption between the prophylaxis treatments (5768.2 [1697.4] and 5197.8 [5005.1] IU kg^−1^ per year for standard and PK-tailored prophylaxis, respectively for the ITT analysis set, which was similar for the PP analysis set). As expected, median (IQR) annualized consumption was significantly less (*P* < 0.0001) during on-demand treatment compared with during prophylaxis (2152.2 [1940.0] and 5733.3 [2929.5] IU kg^−1^ per year).

### Health-related quality of life

Fifty-seven subjects ≥ 14 years of age completed the HRQoL questionnaire at the end of each treatment period. There were no differences in median scores between the two prophylaxis regimens; whereas, statistically significant improvements for the bodily pain domain (4.1; *P* = 0.0007) and PCS (3.6; *P* = 0.0002) were observed at the end of prophylaxis compared with the end of on-demand treatment. These improvements are larger than the established MIDs for these SF-36v1 domains and can therefore be considered clinically significant [20].

### Safety

Safety was assessed in all 73 treated subjects. A total of 200 AEs were reported by 44 subjects: 186 were non-serious events in 41 subjects, of which 19 were considered related to the treatment, and 14 were serious AEs (SAE) in 11 subjects, of which 1 (0.5%) was considered treatment related. No subject developed a confirmed FVIII inhibitor. There were no deaths or withdrawals as a result of AEs. The reported treatment-related SAE was a case of a possible low-titer FVIII inhibitor (0.4 BU mL^−1^), which was unconfirmed, unaccompanied by symptoms of inhibitor presence and disappeared at the subject’s subsequent test. One previously unreported, treatment-related mild AE involved five episodes of palpitations with dizziness in one subject who had several infusions before and after each event without further symptoms. Other AEs considered treatment-related were hematoma at the venipuncture site, chest discomfort, dyspnea, hyperhidrosis, generalized rash, pyrexia and headache. None of the changes in clinically significant laboratory values or vital sign parameters were considered treatment-related. There were no statistically significant differences in mean (± SD) AE rates between standard and PK-tailored prophylaxis treatments (0.356 ± 2.012 vs. 0.089 ± 0.383, respectively).

With no subjects developing a confirmed FVIII inhibitor in the present study and one in the overall rAHF-PFM clinical program, which includes 270 PTPs who had at least 10 exposure days or at least 120 days of observation, the PTP inhibitor incidence is 0.37% [95% confidence interval: 0.02; 2.13%].

## Discussion

The two prophylaxis regimens in the present study demonstrated similar efficacy and safety (i.e. no significant differences in ABRs and AE rates) for the prevention and management of bleeding. Both regimens were targeted to maintain FVIII levels at or above 1% with standard prophylaxis given every second day and PK-tailored prophylaxis given every third day. The utility of prophylaxis dose tailoring with individual PK has focused on optimizing treatment efficacy and FVIII usage with an increased infusion frequency [22]. The results from the present study suggest that the PK-tailored prophylaxis regimen, which used similar amounts of rFVIII and fewer infusions (one less infusion per week), is a viable treatment alternative to standard prophylaxis. The availability of this option could increase treatment adherence, particularly in children and adolescents, for whom compliance with long-term medical regimens is especially challenging.

Because prophylaxis is time consuming and requires direct venipuncture or a central venous catheter, the frequency of infusions has hitherto posed a challenge [15,23]; however, optimal treatment outcomes may be negatively influenced by poor treatment adherence. In a *post hoc* analysis, subjects considered ‘adherent’ with treatment had lower ABRs than those considered ‘not adherent’. Although numbers of ‘non-adherent’ subjects in the present study were small, the result is consistent with previously published findings [24].

As each subject was first treated on-demand for 6 months and then on prophylaxis for 12 months, statistical comparisons between these regimens were paired. Treatment with either prophylaxis regimen significantly (*P* < 0.0001) reduced ABRs for bleeding of all etiologies and types, including hemarthroses, compared with on-demand treatment. These data clearly demonstrate that all bleeding, including hemarthroses, is reduced with prophylaxis compared with on-demand treatment.

After only 12 months of prophylactic treatment, subjects previously treated on-demand (i.e. for at least 12 months before and 6 months during the study) had statistically and clinically significant improvements in physical HRQoL (as measured by the bodily pain domain and PCS) compared with during the on-demand treatment period. These results are probably explained by the significant reduction in ABRs observed while subjects were on prophylaxis and are consistent with the aim of switching adolescents and adults from on-demand treatment to prophylaxis to slow joint deterioration and improve quality of life [12,15,25,26].

The efficacy of treating bleeding episodes (i.e. the number of infusions used and the efficacy ratings) was similar among regimens, which is consistent with results observed previously with rAHF-PFM [5,21,23]. Examination of AEs, laboratory parameters, vital signs and immunogenicity demonstrated that rAHF-PFM was safe and well-tolerated for prophylactic use. Rates of AEs were low considering the long study duration (4.5 years) and subject participation of approximately 1.5 years, and were similar between the two prophylaxis regimens. None of the subjects developed a confirmed FVIII inhibitor.

In conclusion, the demonstrated comparability of standard and PK-tailored prophylaxis regimens in terms of safety and bleeding prevention in hemophilia A patients, indicates that PK-tailored prophylaxis is an effective alternative to the standard regimen with similar amounts of FVIII and one less infusion per week. Compared with on-demand treatment, both prophylaxis regimens significantly reduced bleeding, including spontaneous and traumatic hemarthroses, and improved the quality of life for adolescent and adult patients. The present study further confirms and extends the safety and effectiveness of rAHF-PFM for controlling and preventing bleeding in the management of hemophilia A.

## Addendum

W. Y. Wong, P. Schroth, L. Patrone designed the study; L. A. Valentino, V. Mamonov, A. Hellmann, D. V. Quon, A. Chybicka performed the study; P. Schroth performed the statistical analyses; W. Y. Wong, P. Schroth, L. Patrone analyzed and interpreted data; L. Patrone prepared the manuscript; L. A. Valentino, W. Y. Wong, P. Schroth critically reviewed the manuscript.

## Supporting Information

**Data S1.** Supporting methods, results and tables.
