# Supplementary material for: A randomized comparison of two prophylaxis regimens and a paired comparison of on-demand and prophylaxis treatments in hemophilia A management
Source: J Thromb Haemost. 2012 Mar;10(3):359–67. doi: 10.1111/j.1538-7836.2011.04611.x (PMC3488301; doi:10.1111/j.1538-7836.2011.04611.x)
Supplement: Supplementary file 1 [file jth0010-0359-SD1.doc]

**Supporting Information for (Title):** A randomized comparison of two prophylaxis regimens and a paired comparison of on-demand and prophylaxis treatments in hemophilia A management

**Authors:** Leonard A. Valentino, Vasily Mamonov, Andrzej Hellmann, Doris V. Quon, Alicja Chybicka, Phillip Schroth, Lisa Patrone, and Wing-Yen Wong for the ADVATE Prophylaxis Study Group

# Supporting Study Methods

## Study Design

A summary of the study procedures and assessments is provided in Supporting Info. Table 1. Dosing for the on-demand treatment of hemorrhages was dependent on the severity and type of bleeding (Supporting Info. Table 2). The hemostatic efficacy of treatment was rated by the subject or site staff using a 4-point ordinal scale (Supporting Info. Table 3).

Subject compliance with treatment was monitored by regularly scheduled telephone calls, an electronic compliance tool provided to the subject and direct review of the subject's source data at the sites and evaluation against the protocol requirements. Drug accountability was evaluated at each interval study visit and at study termination by comparing the infusions recorded in the subject diary, empty vials returned by each subject to the site, and the site's dispensing record.

# Supporting Study Results

## Efficacy results

The mean (range) treatment period of the per-protocol (PP) analysis set was 185 days (137 to 254) for the on-demand regimen, 362 days (283 to 397) for standard prophylaxis, and 361 days (287 to 382) for PK-tailored prophylaxis. Over these periods, a total of 1351 hemorrhages occurred in 53 subjects who were treated on-demand, 77 hemorrhages in 17 of 30 subjects treated by standard prophylaxis, and 75 hemorrhages in 14 of 23 subjects treated by PK-tailored prophylaxis. No subject treated on-demand was hemorrhage-free during the 6-month treatment period, whereas 13 of 30 and 9 of 23 subjects treated with standard and PK-tailored prophylaxis, respectively (overall 41.5%) experienced no bleeding during the 12-month prophylaxis period.

ABRs for all etiologies (spontaneous and traumatic) and types of bleeding (joint and non-joint) for standard and PK-tailored prophylaxis were similar, and reductions in ABRs compared to on-demand treatment were also similar (Supporting Info. Table 4).

Supporting Info. Table 1 Schedule of study procedures and assessments

| Procedure/ assessment | | Screening | | Part 1  PK | | | Part 1 and 2 interval study visits^b^ | | Study termination | | |
| --- | --- | --- | --- | --- | --- | --- | --- | --- | --- | --- | --- |
|  |  |  |  | Pre-infusion^a^ | | Post-infusion |  |  | Pre-infusion^a^ | Post-infusion | |
| Informed Consent | | X | |  | |  |  | |  |  | |
| Inclusion/Exclusion | | X | |  | |  |  | |  |  | |
| Medical History | | X | |  | |  |  | |  |  | |
| Interval Medical History | |  | |  | |  | X | | X |  | |
| Physical Exam | | X | |  | |  | X | | X |  | |
| Vital Signs | |  | | X | | X |  | |  |  | |
| Clinical Laboratory Assessments | | X | | X | | X | X | | X | X | |
| Concomitant Medication | | X | |  | | X | X | |  | X | |
| AEs | |  | |  | | X | X | |  | X | |
| Subject Diary | |  | | X | |  | X | | X |  | |
| HRQoL Questionnaire | | X | |  | |  | X | |  | X | |
| Clinical laboratory assessments | | | | | | | | | | | |
| CBC | W | | W | | W | | W | W | | |  |
| Clinical Chemistry | S | | S | | S | | S | S | | |  |
| Antibodies to HIV | S | |  | |  | |  |  | | |  |
| FVIII Activity | P^g^ | | P | | P | | P | P | | | P |
| FVIII Inhibitor | P^g^ | |  | |  | | P | P | | |  |
| INR | W | |  | |  | |  |  | | |  |
| Pregnancy Test | X | |  | |  | | X | X | | |  |
| W, whole blood; S, serum; P, plasma; X, laboratory testing required | | | | | | | | | | | |

Supporting Info. Table 2 rAHF-PFM treatment guidelines during the on-demand period

| Type of bleeding | Dose | Frequency of dosing |
| --- | --- | --- |
| Superficial bleeding and epistaxis, gum bleeding | 10 to 20 IU/kg | Repeat infusions every 12 to 24 hours for 1 to 3 days until the bleed is resolved. |
| Minor hemarthrosis | 20 to 40 IU/kg | Repeat infusions every 12 to 24 hours for 3 days or more until the pain and moderate disability/incapacity are resolved. |
| Moderate hemarthrosis and deep muscle bleed | 30 to 60 IU/kg | Repeat infusions every 12 to 24 hours for 3 days or more until the pain and moderate disability/incapacity are resolved. |
| Major hemarthrosis or life-threatening hemorrhage | 60 to 100 IU/kg | Repeat infusions every 8 to 12 hours until the bleed is resolved. |
| Genitourinary, gastrointestinal and intracranial episode | 60 to 100 IU/kg | Repeat infusions every 8 to 12 hours until the bleed is resolved. |

Supporting Info. Table 3 Hemostatic efficacy rating scale for treatment of bleeding

| Excellent | Full relief of pain and cessation of objective signs of bleeding (e.g., swelling, tenderness, and decreased range of motion in the case of musculoskeletal hemorrhage) within approximately 8 hours of a single infusion. No additional infusion is required for the control of bleeding. Administration of further infusions to maintain hemostasis would not affect this scoring. |
| --- | --- |
| Good | Definite pain relief and/or improvement in signs of bleeding within approximately 8 hours after the infusion. Possibly requires more than 1 infusion for complete resolution. |
| Fair | Probable or slight relief of pain and slight improvement in signs of bleeding within approximately 8 hours after the infusion. Requires more than 1 infusion for complete resolution. |
| None | No improvement or condition worsens. |

Supporting Info. Table 4 Comparison of annualized bleeding rates (ABRs) between treatment regimens*

|  |  | **Standard prophylaxis** | **PK-tailored prophylaxis** |
| --- | --- | --- | --- |
| **All Etiologies, All Types** | Median (IQR) ABR | 1.0 (3.5) | 2.0 (6.9) |
|  | ABR Difference with on-demand^†^ | *P<.0001* | *P<.0001* |
|  | % Reduction with on-demand^‡^ | 98.0 (6.7) | 95.9 (19.8) |
|  | % Reduction difference with on^_^demand^†^ | *P<.0001* | *P<.0001* |
| **All Etiologies, Joint Types** | Median (IQR) ABR | 1.0 (2.1) | 2.0 (5.9) |
|  | ABR Difference with on-demand^†^ | *P<.0001* | *P<.0001* |
|  | % Reduction with on-demand^‡^ | 97.8 (6.2) | 95.8 (19.6) |
|  | % Reduction difference with on^_^demand^†§^ | *P<.0001* | *P<.0001* |
| **All Etiologies, Non-Joint Types** | Median (IQR) ABR | 0 (0) | 0 (0) |
|  | ABR Difference with on-demand^†^ | *P<.0001* | *P<.0001* |
|  | % Reduction with on-demand^‡^ | 100 (2.7) | 100 (5.3) |
|  | % Reduction difference with on^_^demand^†^ | *P<.0001* | *P<.0001* |
| **Spontaneous, All Types** | Median (IQR) ABR | 0 (1.9) | 0 (3.1) |
|  | ABR Difference with on-demand^†^ | *P<.0001* | *P<.0001* |
|  | % Reduction with on-demand^‡^ | 100 (5.6) | 98.7 (8.9) |
|  | % Reduction difference with on^_^demand^†^ | *P<.0001* | *P<.0001* |
| **Spontaneous, Joint Types** | Median (IQR) ABR | 0 (1.6) | 0.5 (3.1) |
|  | ABR Difference with on-demand^†^ | *P<.0001* | *P<.0001* |
|  | % Reduction with on-demand^‡^ | 100 (3.9) | 99.4 (11.4) |
|  | % Reduction difference with on^_^demand^†^ | *P<.0001* | *P<.0001* |
| **Traumatic, All Types** | Median (IQR) ABR | 0 (1.0) | 1.0 (3.8) |
|  | ABR Difference with on-demand^†^ | *P<.0001* | *P<.0001* |
|  | % Reduction with on-demand^‡^ | 100 (5.0) | 87.6 (32.0) |
|  | % Reduction difference with on^_^demand^†^ | *P<.0001* | *P<.0001* |
| **Traumatic, Joint Types** | Median (IQR) ABR | 0 (1.0) | 0.5 (2.0) |
|  | ABR Difference with on-demand^†^ | *P<.0001* | *P<.0001* |
|  | % Reduction with on-demand^‡^ | 100 (4.2) | 90.8 (31.4) |
|  | % Reduction difference with on^_^demand^†^ | *P<.0001* | *P<.0001* |
| ^*^ Intention-to-treat analysis set  ^†^ Paired difference between on-demand and prophylaxis (Wilcoxon signed-rank test)  ^‡^ Median % reduction in ABRs between on-demand and any prophylaxis | | | |
